# *In Situ* Structure of an Intact Lipopolysaccharide-Bound Bacterial Surface Layer

**DOI:** 10.1016/j.cell.2019.12.006

**Published:** 2020-01-23

**Authors:** Andriko von Kügelgen, Haiping Tang, Gail G. Hardy, Danguole Kureisaite-Ciziene, Yves V. Brun, Phillip J. Stansfeld, Carol V. Robinson, Tanmay A.M. Bharat

**Affiliations:** 1 Sir William Dunn School of Pathology, University of Oxford, South Parks Road, Oxford OX1 3RE, United Kingdom; 2 Central Oxford Structural Microscopy and Imaging Centre, South Parks Road, Oxford OX1 3RE, United Kingdom; 3 Physical and Theoretical Chemistry Laboratory, University of Oxford, South Parks Road, Oxford OX1 3TA, United Kingdom; 4 Department of Biology, Indiana University, Bloomington, IN 47405, USA; 5 Structural Studies Division, MRC Laboratory of Molecular Biology, Cambridge CB2 0QH, United Kingdom; 6 Département de microbiologie, infectiologie et immunologie, Université de Montréal, C.P. 6128, Succ. Centre-ville, Montréal, QC H3C 3J7, Canada; 7 Department of Biochemistry, University of Oxford, South Parks Road, Oxford OX1 3QU, United Kingdom

**Keywords:** surface layer, S-layer, bacteria, cryo-EM, cryo-ET, tomography, sub-tomogram averaging, LPS, lipopolysaccharide, *in situ* structural biology

## Abstract

Most bacterial and all archaeal cells are encapsulated by a paracrystalline, protective, and cell-shape-determining proteinaceous surface layer (S-layer). On Gram-negative bacteria, S-layers are anchored to cells via lipopolysaccharide. Here, we report an electron cryomicroscopy structure of the *Caulobacter crescentus* S-layer bound to the O-antigen of lipopolysaccharide. Using native mass spectrometry and molecular dynamics simulations, we deduce the length of the O-antigen on cells and show how lipopolysaccharide binding and S-layer assembly is regulated by calcium. Finally, we present a near-atomic resolution *in situ* structure of the complete S-layer using cellular electron cryotomography, showing S-layer arrangement at the tip of the O-antigen. A complete atomic structure of the S-layer shows the power of cellular tomography for *in situ* structural biology and sheds light on a very abundant class of self-assembling molecules with important roles in prokaryotic physiology with marked potential for synthetic biology and surface-display applications.

## Highlights

• 3.7 Å cryo-EM structure of S-layer bound to O-antigen of lipopolysaccharide (LPS)
• MD simulations and native MS show Ca^2+^-dependent S-layer binding to LPS
• 4.8 Å *in situ* cryo-ET structure of the S-layer on *Caulobacter crescentus* cells
• Integrated structural biology reveals principles of LPS-mediated S-layer assembly

## Graphical Abstract

**Figure:**
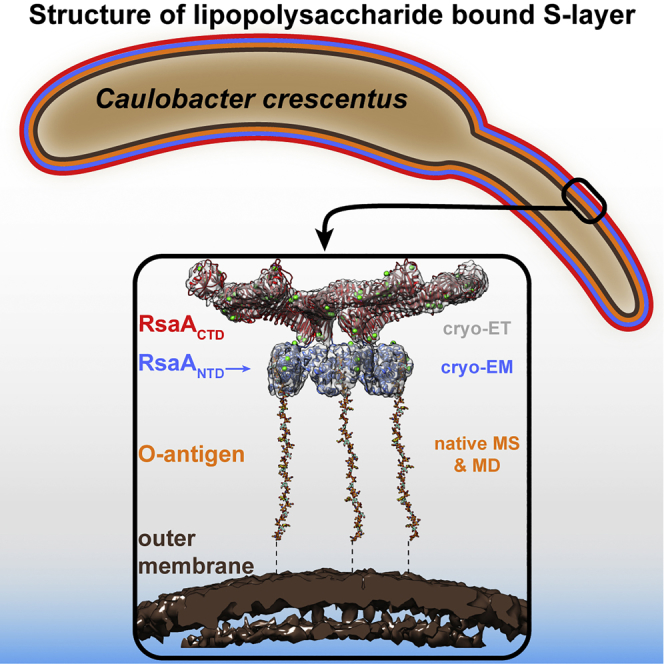

## Teaser

Cryo-electron tomography allows structure determination of the bacterial S-layer in its cellular context at near-atomic resolution, providing insights into how it’s assembled and organized.

## Introduction

Most bacterial and all archaeal cells are encapsulated by a paracrystalline, sheet-like, proteinaceous sheath known as a surface layer (or S-layer) (Sára and Sleytr, 2000). S-layers are made up of two-dimensional lattices built by repeated interactions between a special class of proteins called S-layer proteins (Sleytr et al., 2014). Due to high-copy numbers of S-layer proteins in prokaryotic cells, it is estimated that S-layer proteins are the most abundant class of proteins on earth (Pum et al., 2013). S-layers play critical roles in prokaryotic physiology, ranging from cell-shape determination to protection from predators and phages (Sleytr et al., 2014). Since the first observation of S-layers over half a century ago (Houwink, 1953), structural biology information on S-layers has been scarce because of the inherent difficulty in studying these flexible two-dimensional arrays using the available structural biology techniques. Pioneering electron microscopy investigations have revealed the low-resolution organization of S-layer lattices (Lupas et al., 1994, Smit et al., 1992, Sumper et al., 1990); however, only a few atomic structures of purified S-layer domains have been reported thus far (Arbing et al., 2012, Baranova et al., 2012, Bharat et al., 2017).

Even less is known at the atomic level about how S-layers are anchored and assembled on cells. In archaeal cells, S-layers are often directly bound to the cell membrane (Albers and Meyer, 2011), and Gram-positive bacterial S-layers are buried in the cell wall (Fagan and Fairweather, 2014, Sára and Sleytr, 2000). In Gram-negative bacteria such as *Caulobacter crescentus* or *Campylobacter fetus*, S-layers are retained on cells by lipopolysaccharide (LPS) or endotoxin molecules present in the outer membrane (OM) (Fagan and Fairweather, 2014, Sára and Sleytr, 2000). LPS of Gram-negative bacteria is an abundant glycolipid responsible for bacterial recognition by foreign agents such as the human immune system or bacteriophages. LPS consists of lipid A, core oligosaccharides (OSs), and a repetitive O-antigen polysaccharide (PS) (Cabeen et al., 2010). Recent structural studies on the LPS have focused on O-antigen secretion through the inner membrane (Bi et al., 2018, Caffalette et al., 2019), LPS transport across the periplasm (Li et al., 2019, Owens et al., 2019), and its subsequent secretion through the OM (Dong et al., 2014, Qiao et al., 2014). However, there is limited information on O-antigen structure (Steinbacher et al., 1996); therefore, its native arrangement and conformation on cells remains enigmatic.

To study the structure, anchoring, and assembly of S-layers on LPS molecules in the OM of Gram-negative bacteria, we turned to *C. crescentus*, a well-studied model bacterium with a complex life cycle (Poindexter, 1964). The S-layer of *C. crescentus* is composed of a single, 1026-amino-acid-residue, multi-domain, S-layer protein called RsaA (Smit et al., 1992). We have recently reported the X-ray structure of the C-terminal domain of RsaA (RsaA_CTD_), consisting of residues 250–1026, which form the highly interconnected outer S-layer lattice (Bharat et al., 2017). However, the structure of the N-terminal domain of RsaA (RsaA_NTD_), consisting of residues 1–249, which is a putative LPS-binding domain directly proximal to the OM (Bharat et al., 2017, Ford et al., 2007), is as yet unreported. Therefore, how RsaA is tethered to cells via LPS molecules and the native structure and conformation of LPS on cells beneath the S-layer are all unknown.

Here in this study, we have solved the missing structure of the RsaA_NTD_ bound to the O-antigen of the LPS using single-particle electron cryomicroscopy (cryo-EM). Using native mass spectrometry (MS), we studied the calcium (Ca^2+^) dependence and stoichiometry of sugar binding to RsaA, allowing us to estimate the length of the native O-antigen and understand the architecture of the cellular LPS. Next, molecular dynamics (MDs) simulations combined with electron cryotomography (cryo-ET) allowed us to probe the assembly mechanism of the S-layer on cells. Finally, we used advanced subtomogram averaging techniques to resolve a near-atomic resolution structure of the cellular S-layer, providing unprecedented insights into the S-layer and LPS structure at the *C. crescentus* cell surface. In summary, we report an atomic-level structure of the complete cellular S-layer bound to LPS and multiple Ca^2+^ ions as it is found on the surface of bacterial cells, providing detailed information on S-layer organization and assembly on LPS.

## Results

### Biochemical Reconstitution and Cryo-EM Analysis of RsaA Binding to LPS

**Figure S1:**
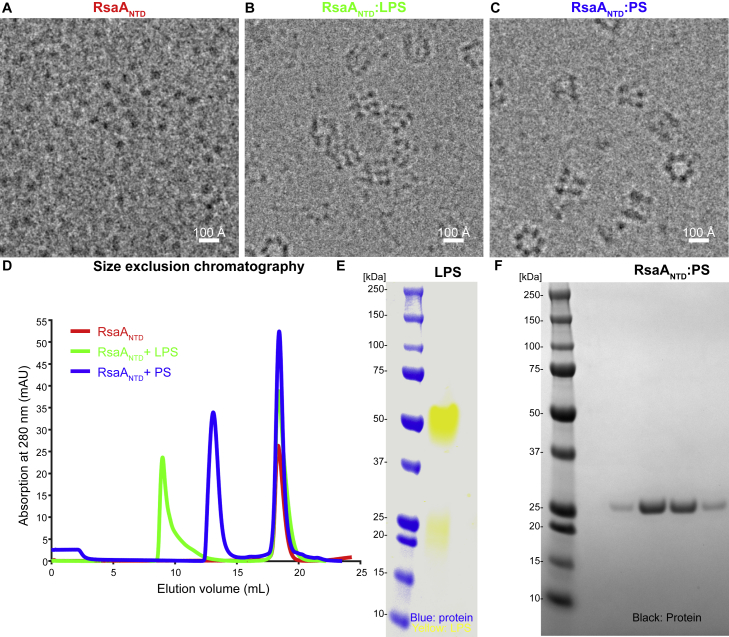
Biochemical Reconstitution of the RsaA_NTD_:PS Complex, Related to Figure 1 (A) Cryo-EM image of purified monomeric RsaA_NTD_. (B) Image of reconstituted oligomeric RsaA_NTD_:crude LPS aggregate complex. (C) Cryo-EM image of purified RsaA_NTD_:PS complex. (D) Gel-filtration profiles of monomeric RsaA_NTD_ (red), RsaA_NTD_ + crude LPS (green) and RsaA_NTD_ + PS (blue) corresponding to images in (A–C). (E) SDS-PAGE of purified crude LPS stained with Pro-Q Emerald 300 (yellow) overlaid with the same gel stained with Coomassie brilliant blue G-250 (blue). (F) SDS-PAGE analysis of purified and mass-spectrometry verified RsaA_NTD_:PS sample stained with Coomassie brilliant blue G-250 (black).

**Figure 1:**
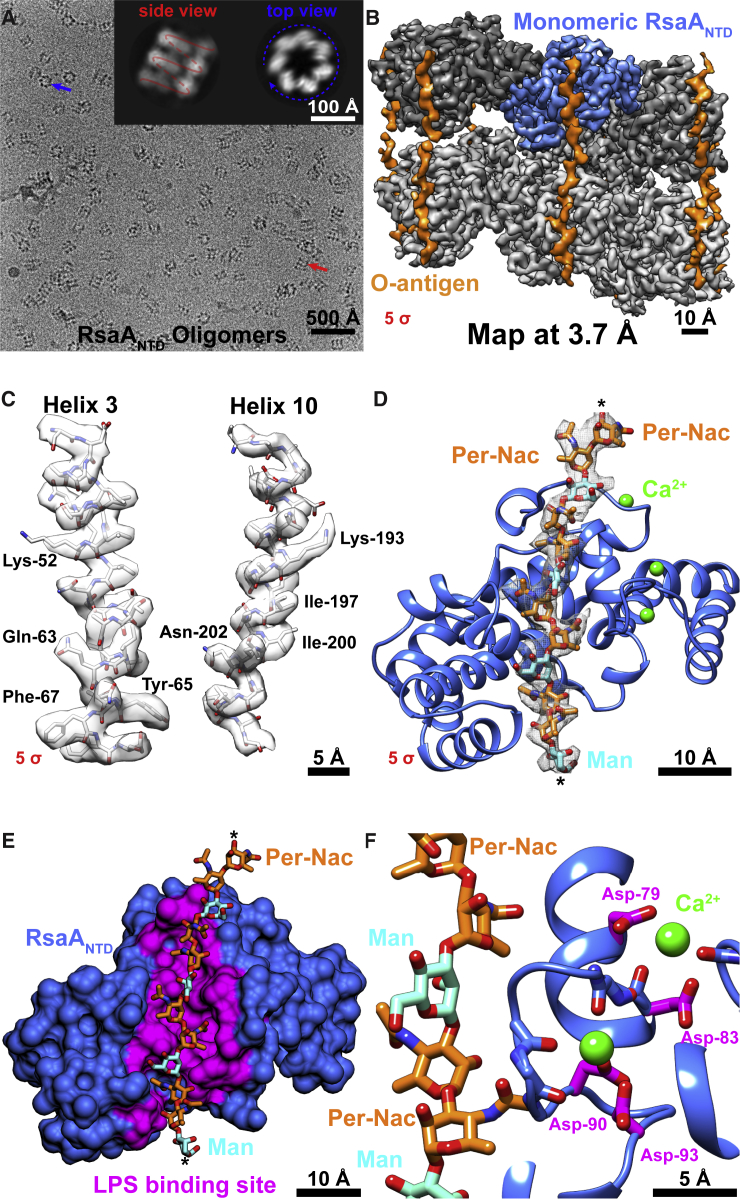
Cryo-EM Reconstruction of the RsaA_NTD_:PS Complex at 3.7 Å Resolution (A) Cryo-EM image of the purified complex. Inset: class averages with the spiral-like nature of the complex highlighted (see Figure S1). (B) Density map of the complex (contour level on the lower left of panel). Different subunits are shown in different shades of gray, and density corresponding to the O-antigen of the LPS is orange (see Video S1). (C) Regions of the map along with the built atomic model showing resolved secondary structure elements and side-chain fits. Due to the α-helical nature of the RsaA_NTD_, the fit of the model to the cryo-EM map is exceptional (see Figure S2). (D) The refined atomic model of a single RsaA_NTD_ subunit from the complex shown as a ribbon diagram. A stick representation of the main chain of the O-antigen is shown within the cryo-EM density. O-antigen chain is continuous along the spiral, denoted by asterisks (^∗^). (E) Surface representation of a single RsaA_NTD_ subunit showing O-antigen binding residues in magenta. (F) Close up of two Ca^2+^ ion binding sites in relation to the O-antigen binding pocket.

To investigate cellular anchoring of RsaA on LPS, we reconstituted RsaA binding to LPS *in vitro* and studied the assembled complex using single-particle cryo-EM. A mutant *C. crescentus* strain that carried a tobacco etch virus (TEV) cleavage site between RsaA_NTD_ and RsaA_CTD_ after position 250 was used to purify RsaA_NTD_ (STAR Methods). Purified RsaA_NTD_ was primarily monomeric in solution and appeared as small particles on cryo-EM grids (Figures S1A and S1D). Next, we purified the *C. crescentus* LPS from a mutant strain that lacked *rsaA* and added it to RsaA_NTD_. Large oligomers of RsaA_NTD_ were formed around aggregates of crude LPS (Figures S1B, S1D, and S1E). To separate RsaA_NTD_ bound to LPS aggregates into homogeneous single particles, we pretreated the purified crude LPS with acetic acid to cleave off lipid A (Jones et al., 2015), yielding partially cleaved PS. On addition of this PS to RsaA_NTD_, an oligomeric complex of approximately 650 kDa was obtained, which appeared as separated single particles on cryo-EM grids (Figures S1C, S1D, and S1F). Different views of the complex were observed, and top views of the complex were reminiscent of the inner domain of the *C. crescentus* S-layer on cells (Figure 1A) (Bharat et al., 2017, Smit et al., 1992).

**Figure S2:**
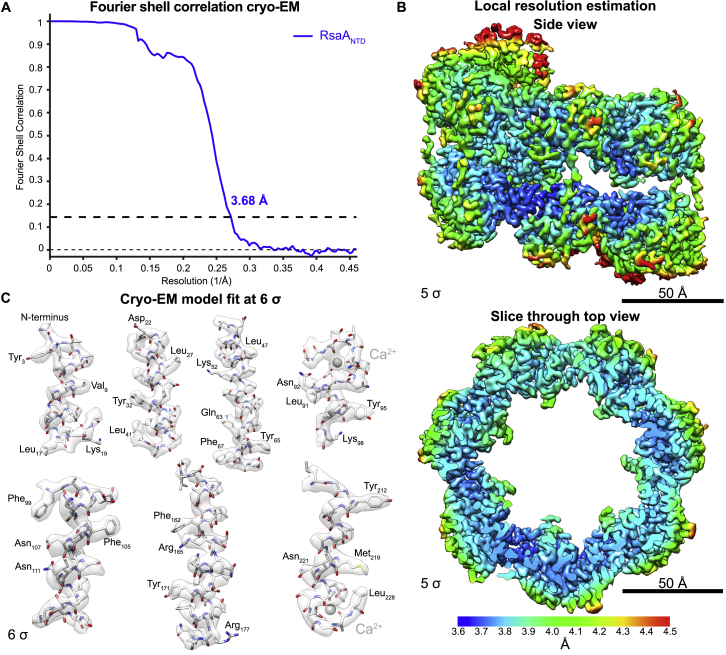
Single-Particle Cryo-EM Reconstruction of the RsaA_NTD_:PS Complex, Related to Figure 1 (A) Fourier shell correlation (FSC) curve of two random half sets of the final reconstructed RsaA_NTD_:PS map shows better than 3.7 Å resolution according to the gold standard criterion of 0.143. (B) Local resolution differences plotted on the cryo-EM density of the RsaA_NTD_:PS complex. (C) Examples of the *de novo* built atomic model fitted into the density contoured at 6 σ away from the mean.

We resolved a density map of the RsaA_NTD_:PS complex at 3.7 Å resolution using single-particle cryo-EM techniques (Scheres, 2012). All secondary structure elements and amino acid side chains were unambiguously assigned, and no significant resolution anisotropy was observed within each RsaA_NTD_ monomer in the cryo-EM map (Figure S2). We used the cryo-EM map to build an atomic model of RsaA_NTD_ along with the bound PS (Figures 1B, 1C, and S2; Video S1; Table S1). In the complex, several copies of RsaA_NTD_ are arranged in a spiral, which is held together along its length by seven chains of PS. Density for the main PS chain of the O-antigen is well resolved (Figure 1D) and agrees with the sequence and linkages reported for *C. crescentus* LPS (Jones et al., 2015). The O-antigen repeating unit is tightly bound to RsaA_NTD_ with several interacting amino acid residues along the length of the protein (Figure 1E). The O-antigen binding pocket is stabilized by functional Ca^2+^ ions that are tightly coordinated to aspartic acid residues in RsaA_NTD_ (Figure 1F), suggesting that Ca^2+^ ions are important for RsaA_NTD_:PS complex assembly.

Video S1. Cryo-EM Structure of the RsaA_NTD_:PS Complex at 3.7 Å Resolution, Related to Figure 1 The reconstructed 3.7 Å resolution cryo-EM density of RsaA_NTD_:PS complex contoured at 5 σ is shown. Individual RsaA_NTD_ subunits are colored in different shades of gray. The O-antigen (orange) is well resolved and forms a connecting density between the successive turns of the spiral-like structure. The *de novo* built atomic model of a single RsaA_NTD_ subunit is fitted into the cryo-EM density where side chains are unambiguously assigned.

### RsaA_NTD_:PS Binding Is Critically Dependent on Ca^2+^ Ions

**Figure 2:**
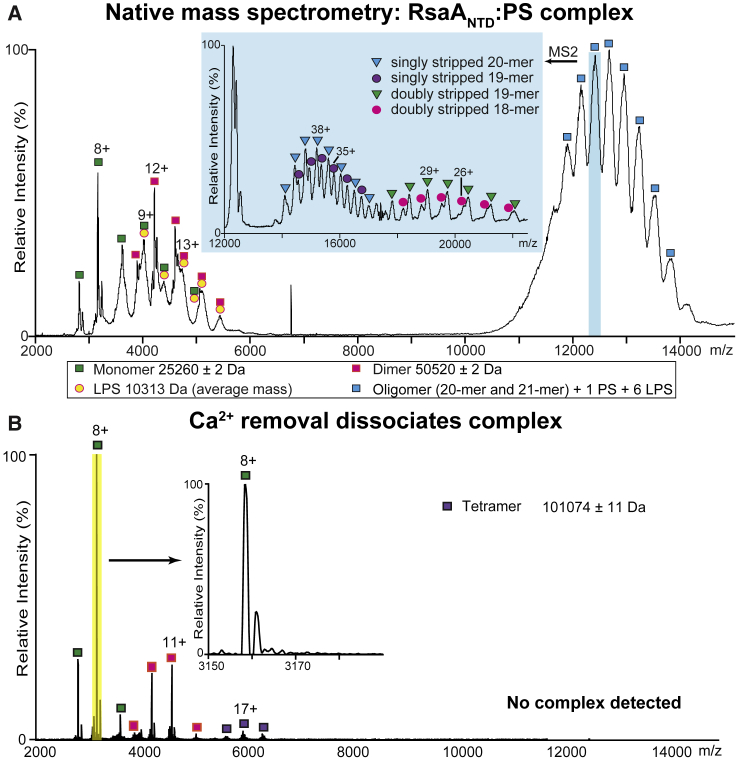
Deducing Protein:Sugar Stoichiometry and Ca^2+^-Dependent Assembly Using High-Resolution Native MS (A) Native mass spectrum of purified RsaA_NTD_:PS complexes show populations of monomer and dimer, both associated with LPS (average mass 10313 Da) and oligomers (20-mer and 21-mer) bound to one unit of PS and six units of LPS. Inset: High energy MS/MS of the oligomeric RsaA_NTD_ performed by isolating the peak at ~12404 *m/z* and dissociating at a voltage of 220V applied to the higher-energy collisional dissociation (HCD) cell. The inset spectrum (blue background) shows stripped oligomers generated by loss of single subunits from the parent complex (21-mer→20-mer→19-mer) and (20-mer→19-mer→18-mer) allowing us to conclude that the original oligomer consists predominantly of a 21-mer and a 20-mer with one unit of PS and six units of LPS each (see Table 1), although other LPS or PS hydrolysis products may also be present. (B) Mass spectrum of the RsaA_NTD_:PS sample after Ca^2+^ removal shows presence of RsaA_NTD_ monomers, dimers, and tetramers only (see also Figure S3).

**Figure S3:**
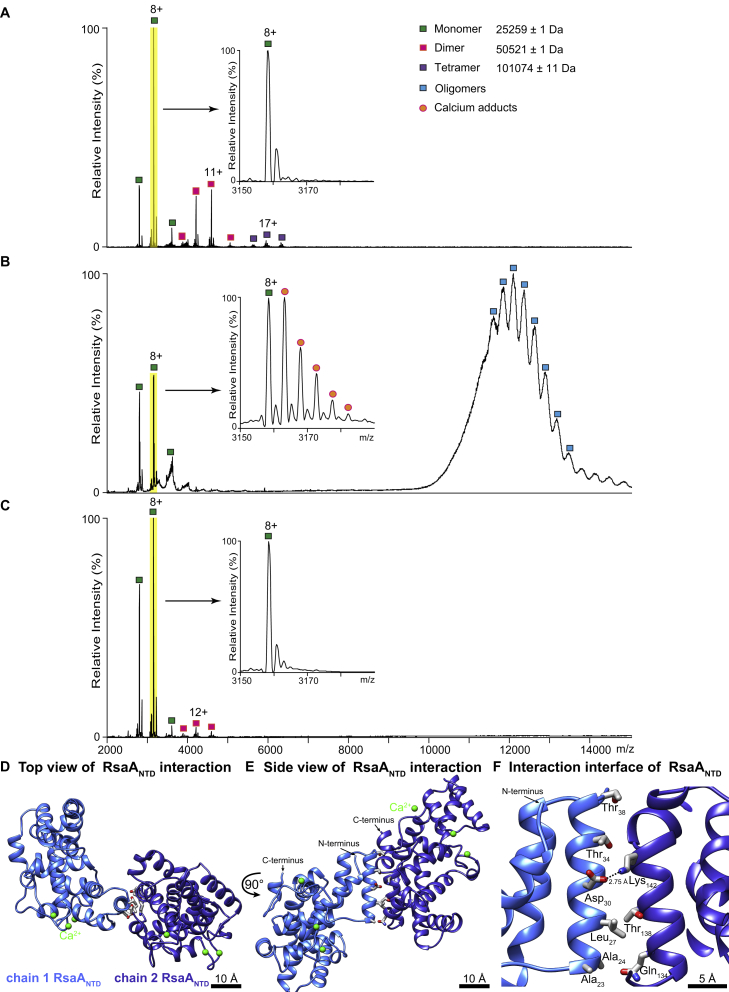
Investigation of the Effect of Ca^2+^ on RsaA_NTD_ Oligomerisation, Related to Figure 2 (A) Mass spectrum of the RsaA_NTD_:PS sample after Ca^2+^ removal shows presence of RsaA_NTD_ monomers, dimers, and tetramers only. Panel same as Figure 2B, shown here for clarity. (B) Mass spectrum of the above sample following incubation with 1 mM calcium acetate indicates that Ca^2+^ ions stimulate the formation of oligomers (RsaA_NTD_:PS complex). (C) After a second Ca^2+^ removal, the complex falls apart into RsaA_NTD_ monomers and dimers. (D) Top view of the RsaA_NTD_:RsaA_NTD_ interaction interface in the cryo-EM structure shown as ribbon diagram. A single α-helix of one RsaA_NTD_ subunit forms the interaction interface with the next RsaA_NTD_ subunit. This interaction is duplicated around the spiral or in the native S-layer hexamer, likely giving large net stabilization. (E) A 90° rotated side view of (D) along the axis of the RsaA_NTD_:PS spiral is shown. (F) Close-up view of (E) highlighting key residues at the RsaA_NTD_:RsaA_NTD_ interaction interface. The interface is stabilized by an ionic interaction between Asp_30_ of one RsaA_NTD_ monomer with Lys_142_ of another RsaA_NTD_ molecule.

To further explore whether Ca^2+^ ions influenced the assembly of the RsaA_NTD_:PS complex, we performed a desalting step to remove Ca^2+^ ions. Native MS (Gault et al., 2016) of the resulting sample showed that the RsaA_NTD_:PS complex dissociated into monomers, dimers, and tetramers of RsaA_NTD_ upon Ca^2+^ removal (Figures 2 and S3). Complex dissociation was reversible, and addition of Ca^2+^ resulted in complex reassembly (Figure S3B). This process of complex disassembly and reassembly could be repeated multiple times (Figure S3C). In the case of the original, assembled RsaA_NTD_:PS complex, native MS showed a series of peaks corresponding to RsaA_NTD_ monomers and dimers, both associated with LPS (Figure 2A), as well as a series of peaks at *m/z* ∼12,000, which were difficult to assign (Figure 2A). Tandem MS indicated that this complex consisted of two oligomeric states, predominantly a RsaA_NTD_ 21-mer, together with a 20-mer. Both oligomers bound preferentially to one unit of PS and six units of full LPS (Figure 2A; Table 1). Although we cannot rule out other PS or LPS stoichiometries, this species predominates and is validated by the cryo-EM reconstruction of the complex that showed seven sugar chains bound to 20 or 21 copies of RsaA_NTD_. In line with the strong tendency to oligomerize observed in native MS, the cryo-EM structure additionally shows an extensive RsaA_NTD_:RsaA_NTD_ oligomerization interface in the complex. This interface is formed by salt bridges between adjoining RsaA_NTD_ subunits. This binding interface is repeated around the spiral (Figures S3D–S3F), likely stabilizing the oligomeric complex.

**Table 1:** Expected and Measured Masses of RsaA_NTD_ Detected in Native MS

Subunit/Complex	Expected Mass (Da)	Measured Mass (Da)	Mass Difference (Da)	Mass Difference (%)
Monomer^a^	25261	25260 ± 2	1	0.003958671
Dimer	50522	50520 ± 2	2	0.003958671
Monomer + LPS (Jones et al., 2015, Smit et al., 2008)	35644.51	35549 ± 519	95.51	0.267951502
Dimer + LPS	60905.51	60857 ± 918	48.51	0.079647966
Singly stripped 20-mer + 1 PS + 6 LPS	577382.22^b^	577492 ± 414	109.78	0.019013402
Singly stripped 19-mer + 1 PS + 6 LPS	552055.22^b^	552599 ± 400	543.78	0.098501016
Doubly stripped 19-mer + 1 PS + 6 LPS	552055.22^b^	552317 ± 190	261.78	0.047419169
Doubly stripped 18-mer + 1 PS + 6 LPS	526728.22^b^	527661 ± 442	932.78	0.17708943

a Residues 2–250 and the amino acids Glu-Asn of the genetically engineered TEV protease site.

b Salt adducts (Na^+^/Ca^2+^) were observed bound to each monomer (average mass of 66 Da per monomer) and were therefore included when we calculated the expected mass of the stripped oligomers. The expected mass of LPS is 10383.51 Da and the expected mass of PS is 8541.16 Da according to the structure of LPS of *C*. *crescentus* reported in a previous study (Jones, 2015).

### RsaA_NTD_ Binds to the O-Antigen of LPS in Two Distinct Ways

**Figure 3:**
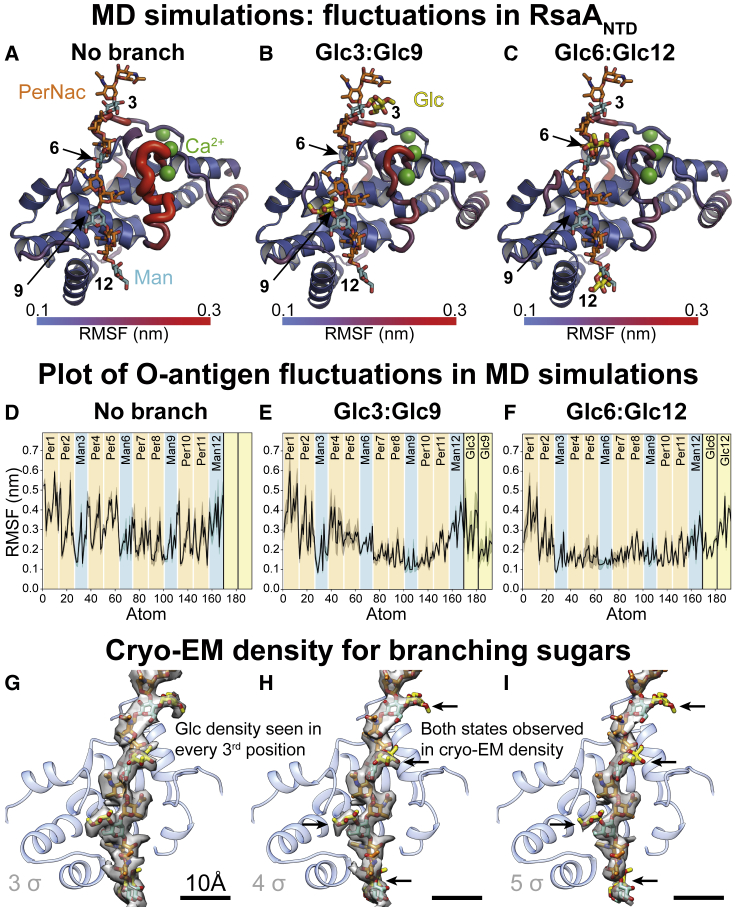
Probing RsaA_NTD_ Binding to the O-Antigen of LPS Using MD Simulations (A) MD simulation of RsaA_NTD_ bound to the O-antigen with no branching sugar moieties. (B) Simulation of RsaA_NTD_ bound to the O-antigen with 3-O-methyl-glucose (Glc) moieties at positions 3 and 9. (C) Simulation of RsaA_NTD_ bound to the O-antigen with Glc moieties at positions 6 and 12. (D) Plot of RMSF of the O-antigen atoms in the MD simulation presented in (A). (E) Plot of RMSF of the O-antigen atoms in the MD simulation presented in (B). (F) Plot of RMSF of the O-antigen atoms in the MD simulation presented in (C). (G–I) Cryo-EM density at different isosurface contour levels showing density for the branching sugar moieties at every third position (see Figure S4)

**Figure S4:**
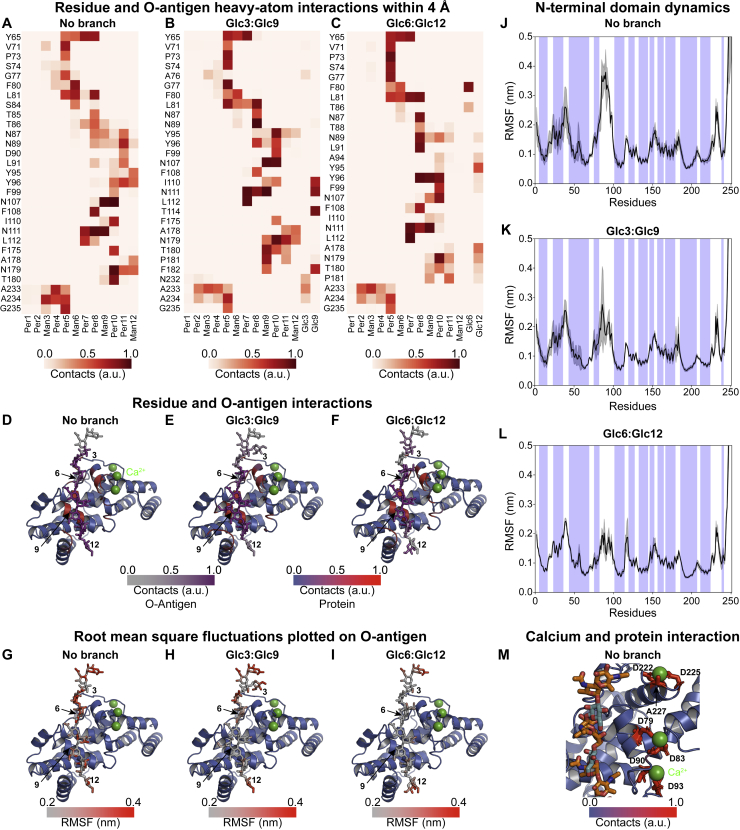
MD Simulations of RsaA_NTD_ Binding to O-antigen, Related to Figure 3 (A) Plot of amino acid residue and O-antigen (heavy-atom) interactions within 4 Å over the course of three 100 ns simulations. The protein-sugar interactions are normalized to 1 (brown), where 0 (white) relates to no contacts. (B) Interaction plot of protein residues and O-antigen with branching Glc moieties at the positions 3 and 9. (C) Protein and O-antigen interactions with branching Glc moieties at the positions 6 and 12. (D–F) Protein and O-antigen interactions from (A–C) are plotted on the ribbon diagram of the protein on a blue to red scale and on the O-antigen on a gray to purple scale. (G–I) Root mean square fluctuations (RMSF) of the O-antigen are displayed on the O-antigen stick diagram on a gray to red color scale (corresponding representation to data in Figures 3D–3F). (J–L) Plot of RMSF of the RsaA_NTD_ residues (see Figures 3A–3C), showing stabilization of the Ca^2+^ binding loop (residues 77–100) by the branching Glc moieties over the course of three 100 ns simulations (α-helical residues in blue background). (M) Interaction of protein residues with Ca^2+^ ions during MD simulations shown on a blue to red scale. All Ca^2+^ ions are stabilized by two aspartic acid residues and backbone carbonyl oxygens in the simulations, as well as in our cryo-EM structure.

Next, we examined the biochemical basis for the O-antigen and RsaA_NTD_ interaction observed in our cryo-EM structure. The repeating unit of the *C. crescentus* O-antigen contains six hexose moieties in the main PS chain: N-acetyl perosamine(PerNac)-PerNac-mannose(Man)-PerNac-PerNac-Man, with a branching 3-O-methyl glucose (Glc) on every sixth position (Jones et al., 2015). While the main PS chain was easily traceable in our map, the position of the branching Glc moiety bound to every sixth Man moiety was not immediately obvious. To help assign the position of the branching Glc in our cryo-EM map, we subjected monomeric RsaA_NTD_ with two repeats of the O-antigen resolved in our structure to MD simulations. Simulations in the absence of branching Glc moieties showed large root mean square fluctuations (RMSFs) for both the sugar and the protein atoms (Figures 3A, 3D, and S4). On the inclusion of the Glc moiety in positions 3 and 9, these fluctuations were dramatically reduced and showed the core of the repeating sugar locked to its binding pocket within the RsaA_NTD_ fold (Figures 3B and 3E). Surprisingly, a parallel simulation with Glc in positions 6 and 12 showed the same result (Figures 3C and 3F), suggesting that there might be two distinct ways that the O-antigen can bind to RsaA_NTD_ (Video S2). To verify this prediction from simulations with orthogonal evidence, we reinspected our cryo-EM map at lower isosurface contour levels (Figures 3G–3I), where we observed density for Glc at every third position, confirming that both states are present in our cryo-EM data and that the structure we resolved likely contains an average of both states, with seven PS chains bound in different registers to the RsaA_NTD_ spiral.

Video S2. MD Simulations of Monomeric RsaA_NTD_ in Complex with Different Branched O-Antigen, Related to Figure 3 MD simulations (100 ns) of monomeric RsaA_NTD_ (blue) in complex with the O-antigen (orange) unbranched (left) or with Glc (yellow) at positions 3 and 9 (middle) or positions 6 and 12 (right).

Another noteworthy observation from the MD simulations was that all three Ca^2+^ ions in the structure remain tightly and stably bound to RsaA_NTD_ throughout the simulations, consistent with our biochemical and native MS data (Figure 2). In fact, the entire Ca^2+^-binding loop consisting of RsaA amino acid residues 77–100 were significantly stabilized when the branching Glc moieties of the O-antigen were included in the simulations (Figures S4J–S4L), suggesting that Ca^2+^ and LPS binding are linked with each other (Figure S4M) and augment complex assembly in a cooperative manner.

### RsaA Binds the Entire Length of the O-Antigen on Cells

**Figure 4:**
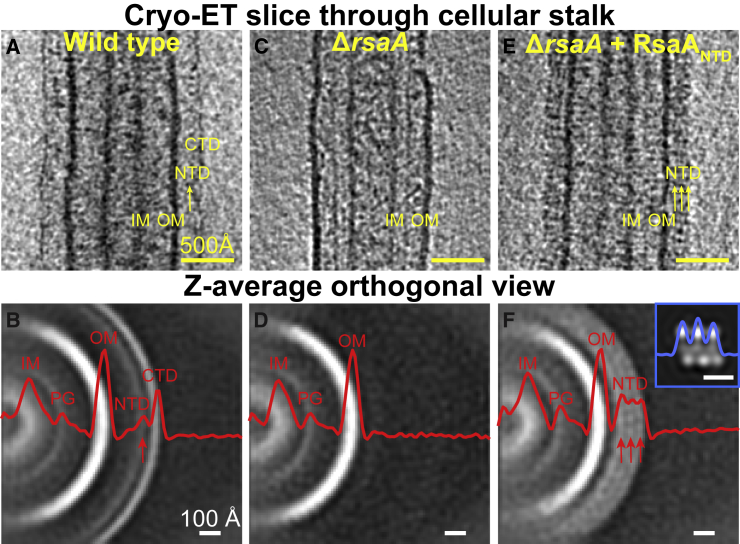
Binding of RsaA_NTD_ to Cellular LPS Occurs along the Entire Length of the O-Antigen (A) Cryo-ET slice through a cellular stalk of CB15N *C. crescentus*. An assembled S-layer is observed, with RsaA_NTD_ and RsaA_CTD_ layers ~180 Å and ~230 Å away from the OM, respectively. (B) Subtomogram averaging of the stalk shows clear densities for the OM, RsaA_NTD_, and RsaA_CTD_. (C) Slice through a stalk lacking RsaA (Δ*rsaA*). (D) Corresponding subtomogram average from Δ*rsaA* stalks. (E) Slice through a Δ*rsaA* cellular stalk with exogenous RsaA_NTD_ added. (F) Corresponding subtomogram average shows three density layers on the outside of the OM. Inset: Side view of the RsaA_NTD_:PS cryo-EM structure also shows three layers of protein bound to the O-antigen PS with the same spacing (see Figure S5).

**Figure S5:**
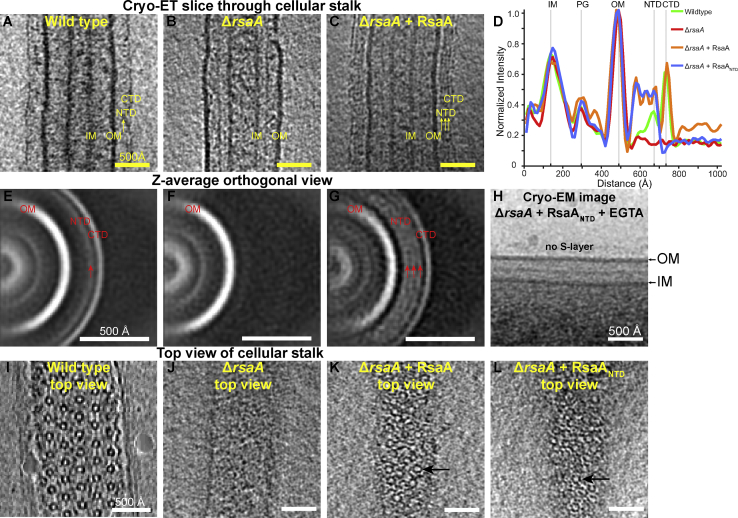
Probing RsaA Binding to Cellular LPS, Related to Figure 4 (A) Cryo-ET slice through a cellular stalk of *C. crescentus*. (B) Slice through a cell stalk lacking RsaA (Δ*rsaA*). (C) Slice through a Δ*rsaA* cell stalk with exogenous full-length RsaA added. Decoration of the LPS in three layers is observed (as in Figures 4E and 4F); however, an additional fourth density layer is observed at the same distance from the OM as the native S-layer RsaA_CTD_. This suggests that RsaA molecules bound to the tip of O-antigen form at least a partial outer S-layer lattice by oligomerization of RsaA_CTD_. (D) Normalized density profiles through subtomogram averages of (A–C) aligned to the OM showing that exogenous added full-length RsaA binds to the entire length of the O-antigen, while forming a partial outer S-layer lattice. (E–G) Corresponding sub-tomogram averages of (A–C) (Figures S5A and S5B; E–G are the same as Figures 4A–4D, shown here for clarity). (H) Cryo-EM image of a Δ*rsaA* cell with exogenous RsaA_NTD_ added together with EGTA. Chelation of Ca^2+^ by EGTA prevents S-layer assembly at the cell surface. (I) Cryo-ET slice through the top of a cell stalk of *C. crescentus* showing a normal, hexagonal S-layer. (J) Slice though the top of a cell stalk lacking RsaA (Δ*rsaA*). (K) Slice through the top of a Δ*rsaA* cell stalk with exogenous full-length RsaA added showing irregularly arranged spiral-like structures (black arrow). (L) Slice through the top of a Δ*rsaA* cell stalk with exogenous RsaA_NTD_ added showing irregularly arranged spiral-like structures (arrow) with characteristics similar to the RsaA_NTD_:PS complex.

Next, we tested whether our *in vitro* results could be replicated on cells. A density for both RsaA_NTD_ as well as RsaA_CTD_ is seen in raw electron cryotomograms of *C. crescentus* cell stalks (Figure 4A) and also in subtomogram averages produced from the surface of these stalks (Figure 4B). Density layers for RsaA domains are absent in a Δ*rsaA* mutant, which does not possess an S-layer (Figures 4C and 4D). To test whether RsaA_NTD_ would also bind to cellular LPS, we added purified RsaA_NTD_ to Δ*rsaA* cells. Instead of forming a single layer of density outside the OM like the native S-layer, we observed three density layers proximal to the OM (Figures 4E and 4F). These density layers had the same appearance and spacing as those seen in side views of our cryo-EM structure of the RsaA_NTD_:PS complex (Figure 4F, inset). This showed that not only can RsaA_NTD_ bind to cellular LPS but also that binding occurs all along the LPS O-antigen due to its repeating nature. Similar results were obtained on the addition of full-length RsaA, where three density layers were observed on top of a RsaA_CTD_ layer (Figure S5). Also consistent with our biochemical data, when Ca^2+^ ions were depleted from the reaction by the addition of EGTA (ethylene glycol-bis(β-aminoethyl ether)-*N*,*N*,*N*′,*N*′-tetraacetic acid) and RsaA_NTD_ was added to Δ*rsaA* cells (Figure S5H), no S-layer assembly or decoration of the cellular LPS in the OM was observed, in line with the desalting experiments conducted in native MS.

In light of these *in situ* results, together with the stoichiometry of the RsaA_NTD_:PS complex observed in cryo-EM and confirmed by native MS in which 20 or 21 monomers of RsaA_NTD_ were observed in the complex bound to seven O-antigen molecules, we predict using the total mass of the complex that the full RsaA_NTD_ spiral would bind on average 294 hexose moieties, which allows us to calculate that there are 42 hexose moieties in the cellular O-antigen of the native *C. crescentus*. This calculation is also consistent with the distances measured between the OM and the S-layer in our cryo-ET data (Figure 4), accounting for the size of the core OS proximal to the OM.

### Near-Atomic Resolution *In Situ* Structure of the *C. crescentus* S-Layer

**Figure S6:**
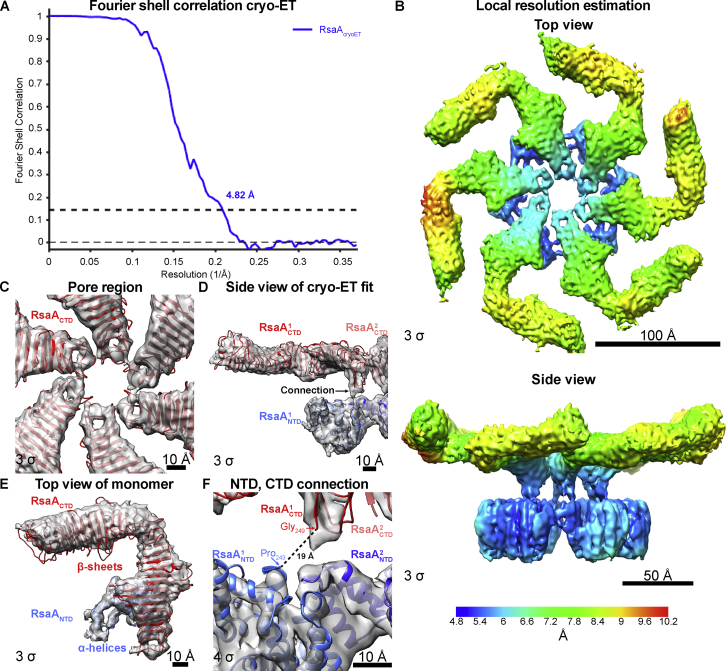
Structure of the Native *C. crescentus* S-Layer Determined by Subtomogram Averaging, Related to Figure 5 (A) Fourier shell correlation (FSC) curve of two half sets of the final reconstructed native RsaA S-layer at a 4.82 Å resolution according to the 0.143 criterion. (B) Local resolution differences plotted on the cryo-ET density showing resolution anisotropy between RsaA_NTD_ and RsaA_CTD_. (C) Atomic models docked into the cryo-ET density (gray) (contour levels on lower left side of panel). The RsaA_CTD_ X-ray structure (PDB: ID 5N8P) fits the central pore region exceptionally well. (D) A side view cross-section of the isosurface (gray) is shown with the docked RsaA_CTD_ X-ray structure (red) and the RsaA_NTD_ cryo-EM structure (blue). RsaA_CTD_ is connected to RsaA_NTD_ by a small linker region. (E) A single monomer of the native RsaA S-layer is shown as top view as in Figure 5B. (F) Close up view of the connecting region as shown in (D) highlights the exceptional model fit of both domains. The C terminus of the solved cryo-EM structure (Pro_243_) is ~19 Å away from the N terminus (Gly_249_) of the X-ray structure (red). The linker region consisting of five residues is poorly resolved indicating flexibility.

**Figure 5:**
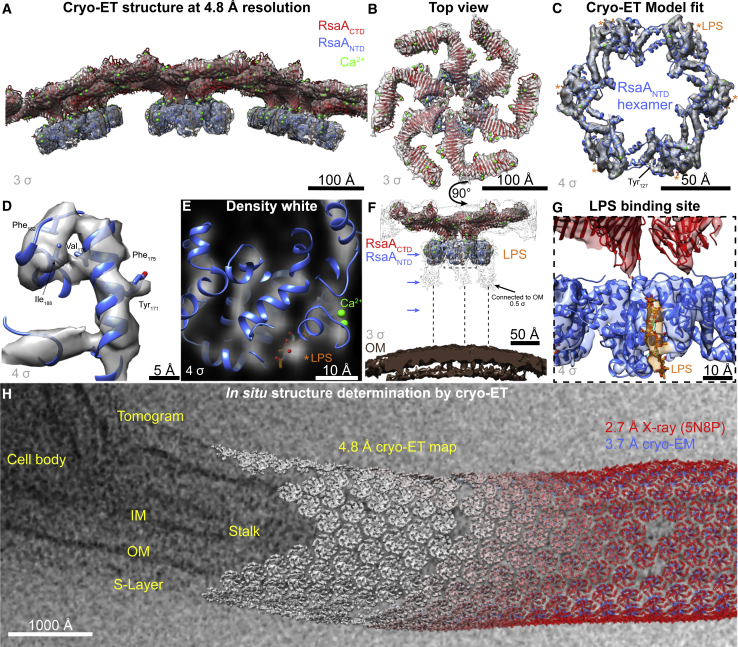
*In Situ* Cryo-ET of the Native *C. crescentus* S-Layer at 4.8 Å Resolution (A) Subtomogram averaging at 4.8 Å resolution of the native S-layer from cell stalks. RsaA_CTD_ X-ray structure (PDB: 5N8P, red ribbon) and RsaA_NTD_ cryo-EM structure (blue ribbon) are docked into the density (contour level shown on lower left of each panel, see Figure S6). (B) Top View of a Single Hexameric Unit (C) Six subunits of the cryo-EM structure of RsaA_NTD_ docked into the cryo-ET map. LPS densities highlighted with asterisks (^∗^). (D) A closeup of a single RsaA_NTD_ α-helix showing some resolved bulky side chains in the cryo-ET map. (E) A ribbon diagram of one RsaA_NTD_ subunit overlaid on a slice through the cryo-ET map. A clear density for the O-antigen is observed at the same relative location as in the cryo-EM RsaA_NTD_:PS structure. (F) A side view of a single hexamer is shown relative to the OM of the cell. Densities of O-antigen bound to RsaA extend downward to the OM (black outline density). Positions of RsaA_NTD_:PS density layers as seen in Figure 4F are highlighted with blue arrows (see Video S3). (G) A closeup view of the O-antigen binding pocket resolved in the cryo-ET map. (H) Cellular structural biology from cells to atoms. Tomographic slice of a *C. crescentus* cell. Copies of the 4.8 Å cryo-ET structure are overlaid on the tomographic slice at their refined cellular locations. Atomic structures determined by X-ray crystallography (RsaA_CTD_ 2.7 Å) and cryo-EM (RsaA_NTD_ 3.7 Å) are docked into the cryo-ET map (see Video S5).

To obtain a more detailed view of the cellular S-layer, we used recently described subtomogram averaging algorithms (Turoňová et al., 2017) to obtain a 4.8 Å resolution structure of the S-layer lattice directly on the surface of cell stalks (Figure S6). The subtomogram averaging map shows large anisotropy in resolution, with the resolution highest in RsaA_NTD_, close to the 6-fold symmetrization axis (Figure S6B). Both the RsaA_NTD_ cryo-EM structure as well as the RsaA_CTD_ X-ray structure (Bharat et al., 2017) could be docked into the cryo-ET density unambiguously as rigid bodies (Figures 5A and 5B; Video S3). All α helices of RsaA_NTD_ are resolved in the cryo-ET map (Figure 5C), and large side chains are resolved, which fit the density well without any further refinement (Figure 5D). Satisfyingly, a clear density for the O-antigen of the LPS is observed in the same relative location as in the single-particle cryo-EM map of the RsaA_NTD_:PS complex (Figure 5E). It is worth mentioning that the RsaA_NTD_:RsaA_NTD_ interface is almost unchanged from the interaction observed in the RsaA_NTD_:PS spiral complex (Figures 1 and S3D–S3F). This observation suggests that the planar hexameric arrangement of RsaA_NTD_ in the native S-layer is at least partially imposed by the hexagonal symmetry of the RsaA_CTD_ outer lattice.

Video S3. *In Situ* Cryo-ET Reconstruction of the Native *C. crescentus* S-Layer at 4.8 Å Resolution, Related to Figure 5 The 4.8 Å cryo-ET map of the native *C. crescentus* S-layer (gray) contoured at 3 σ away from the mean is shown with the docked 2.7 Å X-ray structure (PDB: 5N8P) of RsaA_CTD_ (red). A side view of the structure highlights the RsaA_NTD_ (blue). A close-up view of RsaA_NTD_ shows clear density corresponding to the bound O-antigen. Decreased contour level (0.5 σ) of the cryo-ET map shows that densities of O-antigen bound to RsaA extending downwards toward the OM of the bacterial cell.

There are very few contact sites observed between RsaA_NTD_ and RsaA_CTD_ (Video S4), showing why the two domains can oligomerize independently. The two domains are connected by a short linker region, which appears as a smeared density in the cryo-ET map, suggesting some flexibility ( Figures S6D–S6F; Video S4). The density for the LPS O-antigen is clearly resolved near the RsaA_NTD_ domain, and this density ends within the RsaA_NTD_ layer and does not extend outward to the RsaA_CTD_ (Figure 5G). This indicates that upon secretion and assembly into an S-layer, RsaA proteins localize at the very tip of the O-antigen. These observations are consistent with the fact that antibodies against the O-antigen are unable to stain the O-antigen when the S-layer is present (Walker et al., 1994).

Video S4. Ca^2+^ Ion Stabilization and LPS Tethering of Native *C. crescentus* S-Layer, Related to Figure 5 The outer domain of RsaA (RsaA_CTD_, red) is plotted as a hexagonal lattice determined by subtomogram averaging of the native *C. crescentus* S-layer. A closeup view of the Ca^2+^ dependent outer lattice highlights the dimeric and trimeric interfaces of individual subunits forming the S-layer. A side view of the structure with the RsaA_NTD_ (blue) bound to the O-antigen of LPS, which is stabilized by a Ca^2+^ dependent loop region proximal to the sugar binding site. The RsaA_NTD_ and RsaA_CTD_ domains are connected by a small linker region near residue Pro_243_ of the solved 3.7 Å cryo-EM structure and residue Gly_249_ of the 2.7 Å X-ray structure (Bharat et al., 2017).

**Figure S7:**
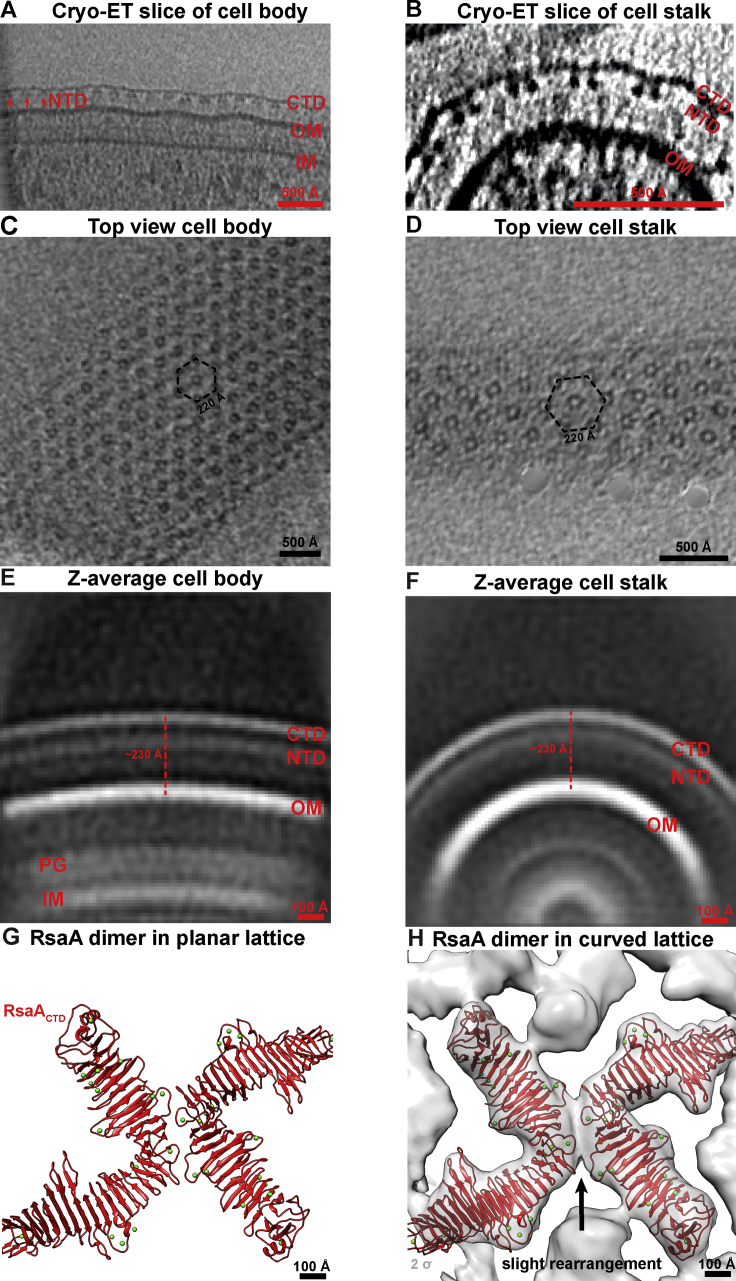
Overall S-Layer Arrangement on the Flat Cell Body and Highly Curved Cell Stalk Is the Same, Related to Figure 6 (A) A cryo-ET slice through the side of a *C. crescentus* cell body (protein density black in all raw cryo-ET slices). The OM is clearly decorated with a S-layer made up of RsaA_NTD_ and RsaA_CTD_ layers (marked). (B) Cryo-ET slice through the tip of a *C. crescentus* cell stalk. The highly curved OM is covered by a S-layer, consisting of the same RsaA_NTD_ and RsaA_CTD_ layers, with same ultrastructural morphology as the S-layer on the cell body. (C) Cryo-ET slice through the top surface of a *C. crescentus* cell body. The near hexagonal planar arrangement of the S-layer with a hexamer:hexamer distance of 220 Å is seen, as shown previously (Bharat et al., 2017) and confirmed in this study. (D) Cryo-ET slice through the top of a *C. crescentus* cell stalk. Although the S-layer lattice is highly curved around the stalk, the pseudo-hexagonal arrangement of the S-layer with a hexamer:hexamer distance of 220 Å is observed, same as the cell body. (E) Despite considerably increased specimen thickness, we performed subtomogram averaging of the outer surface of the *C. crescentus* cell body, which shows clear densities for the OM, RsaA_NTD_, and RsaA_CTD_ (protein density white in all averages). The distance between the OM and the RsaA_CTD_ layer is ~230 Å. (F) Subtomogram averaging of the cell stalk (as shown in Figure 4B) shows a highly curved OM, surrounded by a S-layer. The distance between the OM and the RsaA_CTD_ layer is ~230 Å, same as the cell body, indicating the length of the LPS underneath the S-layer is the same between the stalk and the cell body. (G) The dimeric RsaA_CTD_ interface observed in the outer S-layer lattice in flat planar sheets, solved by X-ray crystallography (PDB: 5N8P). (H) Two copies of RsaA_CTD_ were fitted separately into a subtomogram averaging map produced from curved cell stalks with a large box size to visualize the hexamer:hexamer interfaces. The fit shows a mismatch between the X-ray structure and the subtomogram averaging map, suggesting rearrangement of residues at the dimeric interface concurrent with lattice curvature.

In the other direction toward the cell, at low contour levels, the O-antigen density extends down toward the OM and is probably formed of flexible and fluctuating O-antigen repeats connected to the core OS (Figure 5F; Video S5). The relative distance between the S-layer and the OM remains constant over the cell body and stalks (Figure S7), showing that the length of the LPS O-antigen is the same all around the cell. Furthermore, the S-layer arrangement including the near-hexagonal symmetry is also seen over the cell body (Figure S7). Therefore, the near-atomic resolution *in situ* structure of the *C. crescentus* S-layer allows positioning of not only the two RsaA domains but also the O-antigen of LPS relative to the OM of the bacterial cell (Figure 5H), providing an atomic resolution snapshot of the outermost layer of *C. crescentus* bacteria made up of protein and polysaccharide.

**Figure 6:**
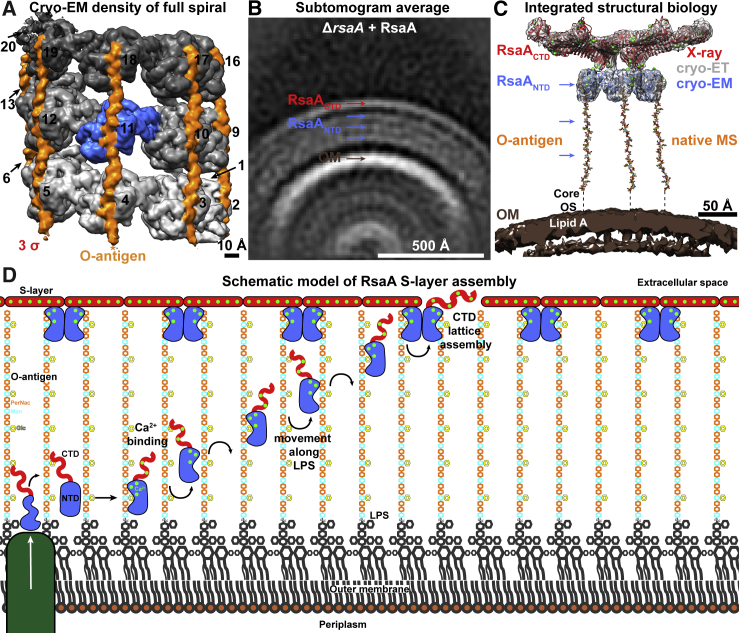
Schematic Model of *C. crescentus* S-Layer Assembly on the Cell Surface (A) Density of the cryo-EM structure of the isolated RsaA_NTD_:PS complex contoured at 3 σ away from the mean. The reconstruction of the entire spiral is shown, no mask applied (3.9 Å resolution map). RsaA_NTD_ subunits are shown in different shades of gray (additionally numbered), and density corresponding to the O-antigen of the LPS is shown in orange. Asterisk (^∗^) denotes density for LPS extending at lower contour levels only in one direction. (B) Subtomogram averaging of the sample with exogenous added full-length RsaA to cells lacking native S-layer (Δ*rsaA*) demonstrates that full-length RsaA can bind along the entire length of the O-antigen and can form a partly assembled outer lattice (panel same as Figure S5G). We expect that only RsaA molecules at the tip of the O-antigen are able to partially assemble the outer lattice because of steric hindrance by a mesh of LPS in the layers below. (C) Using a combined structural approach, X-ray crystallography (RsaA_CTD_, PDB: 5N8P, red), cryo-EM (RsaA_NTD_, blue), cryo-ET (subtomogram average, gray), and native MS (O-antigen, orange), we report a model of a full bacterial S-layer bound to LPS. (D) Schematic model of *C. crescentus* S-layer assembly. RsaA is secreted to the extracellular milieu, where RsaA binds to Ca^2+^ and LPS. This binding has been observed in our cryo-EM structure of the RsaA_NTD_:PS complex (Figure 1) and verified by native MS (Figure 2). Next, RsaA is guided on LPS molecules by binding to the O-antigen along multiple sites, as observed in our cryo-EM structure (Figure 1), confirmed by MD simulations (Figures 3 and S4) as well as by *in situ* experiments showing binding of RsaA along the entire length of the LPS O-antigen (Figure 4). RsaA molecules are unable to assemble into an S-layer lattice near the OM (Figure S5), likely due to steric hindrance by a meshwork of LPS molecules. At the tips of the LPS O-antigen, whose length we accurately estimated using native MS (Figure 2), RsaA molecules can bind with a pre-existing S-layer to complete gaps in the lattice via oligomerization through RsaA_CTD_ (B and Bharat et al., 2017).

Video S5. High-Resolution *In Situ* Structure of the *C. crescentus* S-layer Plotted on the Cells, Related to Figures 5 and 6 Sequential Z-slices through a tomogram of a *C. crescentus* cell illustrating the cellular morphology (yellow annotation). Sub-tomogram averaging of the S-layer on the cell stalks yielded a 4.8 Å map of the native S-layer as well as the cellular localisation of the repeating hexameric units on the OM. A close up view of a single hexameric S-layer unit with fits of the atomic structures of the RsaA_CTD_ (2.7 Å X-ray structure, red) and RsaA_NTD_ (3.7 Å cryo-EM, blue) into the *in situ* cryo-ET map (4.8 Å sub-tomogram averaging, gray) contoured at 3 σ away from the mean. The O-antigen binding site of the RsaA_NTD_:PS structure is present in the native S-layer and the density corresponding to the O-antigen is resolved. At lower isosurface contour levels (3–0.5 σ) densities emanating from the S-layer toward the OM are clearly seen.

## Discussion

One of the outstanding goals of structural biology is to resolve atomic resolution cellular structures and to study cellular processes in atomic detail *in situ*. To this end on the technical side, this study demonstrates how cryo-ET and subtomogram averaging (4.8 Å cellular S-layer) is closing the gap to single-particle cryo-EM (3.7 Å RsaA_NTD_:PS complex) and X-ray crystallography (2.7 Å RsaA_CTD_). Modern cryo-ET imaging can provide molecular resolution insights into the architecture and organization of cells (Chang et al., 2016, Mahamid et al., 2016). Here we used cryo-ET combined with subtomogram averaging to reveal a repeated cellular structure at near-atomic resolution, allowing the fundamental biological process of S-layer assembly to be studied directly in its cellular context (Figure 5H).

Our structure of the complete *C. crescentus* S-layer, together with the O-antigen of the LPS, is a potential target for synthetic biology applications, in which tagged S-layers could be used to link genotype to protein displayed at high copy numbers on surfaces. The first such technical application, developed using the X-ray structure of RsaA_CTD_ has been recently reported (Charrier et al., 2019).

Our structure of the RsaA_NTD_:PS complex confirms previous studies that showed that the N-terminal residues of RsaA are critical for S-layer anchoring (Ford et al., 2007). A majority of the mutations in RsaA_NTD_, including amino acid exchanges or insertions before position 225 in the amino acid sequence, lead to complete loss of S-layer anchoring (Ford et al., 2007), consistent with the extensive interaction observed between the O-antigen and RsaA_NTD_ in our cryo-EM structure (Figures S4A–S4C). Our integrated structural biology approach has technical implications to the future structural studies of LPS. LPS is nearly ubiquitous in Gram-negative bacteria, and it is found in several important human pathogens. LPS assembly and biogenesis has thus rightly been the focus of several prominent structural biology efforts in the recent past, for example Bi et al., 2018 and Caffalette et al., 2019, to name a few. Here we show that by combining cryo-EM and cryo-ET with native MS, it is possible to understand not only the biochemistry but also the cellular architecture and arrangement of LPS, revealing how LPS molecules are located in the space directly outside Gram-negative *C. crescentus* cells. Using a similar integrated approach of combining EM with MS, LPS molecules in other bacteria may be studied, allowing molecular details of this enigmatic molecule to be revealed.

In *C. crescentus*, future genetic and biochemical studies that mutate and perturb the LPS O-antigen might shed light onto how LPS biogenesis and secretion is coupled with S-layer assembly. Only density for the LPS O-antigen bound to RsaA_NTD_ was resolved in our cryo-EM map; therefore it is still not completely clear how the core oligosaccharide and lipid A of the LPS is arranged on cells. Further structural and cell biology studies in the *C. crescentus* system will be required to understand how the LPS is anchored at the other end, away from the S-layer by the bacterial OM.

It is reasonable to expect that newly secreted RsaA proteins might be guided toward the S-layer lattice along O-antigen chains via binding to RsaA_NTD_ (Figure 6). O-antigen binding is Ca^2+^ dependent (Video S4), and Ca^2+^ levels are higher in the extracellular space (Baines et al., 2000, Jeziorski et al., 2008), enough to trigger spontaneous S-layer assembly (Herrmann et al., 2017). RsaA_NTD_ can bind along the entire length of the O-antigen of the cellular LPS (Figures 6B and S5), consistent with the multiple binding modes observed in our cryo-EM structure and confirmed by MD simulations. When full-length RsaA was added to a *C. crescentus* strain lacking an S-layer, formation of an additional (fourth) density layer at the same distance from the OM as the native RsaA_CTD_ layer was observed, suggesting that RsaA molecules bound at the ends of the LPS had at least partially assembled into an S-layer lattice. This indicates that lattice assembly is only possible when RsaA is bound at the tip of the O-antigen, implying that a meshwork of LPS and other molecules might sterically hinder premature S-layer formation.

RsaA_NTD_ domains in the assembled S-layer are attached to the end of the LPS (Figure 6C), and RsaA_CTD_ domains oligomerise in a Ca^2+^-dependent manner to form the outer S-layer lattice (Figures 6C and 5H; Video S5). Overall, both RsaA_NTD_ and RsaA_CTD_ contain several functionally important Ca^2+^ ions, which have roles in LPS binding as well as lattice assembly. Consistent with this strong Ca^2+^ dependence, cooperative assembly is observed in both RsaA domains, allowing this remarkable two-dimensional array to be assembled on the surface of *C. crescentus* cells (Figure 6D). It is interesting to note that Ca^2+^-dependence has been previously described for other unrelated S-layers from Gram-positive bacteria (Baranova et al., 2012) and archaea (Sumper et al., 1990), perhaps suggesting similar mechanisms of assembly. However, further work will be needed to verify this expectation. In the case of the *C. crescentus* S-layer, it is remarkable how division of labor between different RsaA domains, where RsaA_NTD_ mediates cell anchoring and RsaA_CTD_ forms the lattice layer, allows this remarkable polyprotein to perform multiple tasks on the cell surface to form micron-scale, two-dimensional sheaths on membranes with variable curvature.

Finally, fluorescently tagged S-layers derived from our structure will open the door to studying S-layer dynamics (Charrier et al., 2019, Comerci et al., 2019) with respect to the cell cycle and cell morphogenesis. A recent optical microscopy study on the same system has shown that a new *C. crescentus* S-layer is inserted at the mid-cell or at the cell poles (Comerci et al., 2019). In the light of this spatiotemporal regulation of S-layer morphogenesis, it seems remarkable how molecular rearrangements within the cell control biogenesis and assembly of a two-dimensional lattice 180 Å away from the OM, mediated by long rope-like LPS molecules. Future structural and cell biology studies on this system will reveal how S-layer morphogenesis is coupled with critical cellular processes such as cell elongation, peptidoglycan synthesis, LPS biogenesis, LPS secretion, and cell division, providing insights into the mechanism of how spatial information is relayed from the cytoplasm to the extracellular environment of the cell.

## STAR★Methods

### Key Resources Table

**Table:** 

REAGENT or RESOURCE	SOURCE	IDENTIFIER
**Chemicals, Peptides, and Recombinant Proteins**		

ethylene glycol bis(2-aminoethyl ether)-*N*,*N*,*N*′,*N*′-tetraacetic acid (EGTA)	SigmaAldrich	Cat#E3889-100G
Fiducial gold (FG) 10 nm, 400 μL	CMC Utrecht	N/A

**Critical Commercial Assays**		

Pro-Q Emerald 300 Lipopolysaccharide Gel Stain Kit	Thermo Fisher Scientific	Cat#P20495

**Deposited Data**		

RsaA_NTD_:PS structure	This study	PDB: 6T72
RsaA_NTD_:PS cryo-EM map	This study	EMDB: EMD-10389
RsaA S-layer cryo-ET map	This study	EMDB: EMD-10388

**Experimental Models: Organisms/Strains**		

*Escherichia coli* S17-1	(Simon et al., 1983)	S 17-1
*Escherichia coli* Alpha-select™	Bioline	N/A
*Caulobacter crescentus* strain CB15N (NA1000)	(Evinger and Agabian, 1977)	N/A
*Caulobacter crescentus* strain YB5754	E. Quardokus, personal communication	YB5754
*Caulobacter crescentus* strain YB1001	This study	YB1001

**Recombinant DNA**		

pNPTS138	Brun lab collection	N/A
pNPTS138::*rsaA*TEV250	This study	N/A
pNPTS138Δ*rsaA*	(Hardy et al., 2010)	N/A

**Oligonucleotides**		

Primers for generation of *rsaA*TEV250 strain (see Table S2 for details)	This study	N/A
Primers for sequencing of mutant *rsaA*TEV250 strain (see Table S2 for details)	This study	N/A
Primers for PCR and sequencing of mutant Δ*rsaA* strain (see Table S2 for details)	This study	N/A
Primers for sequencing mutant plasmid vectors (see Table S2 for details)	This study	N/A

**Software and Algorithms**		

CCP-EM	(Burnley et al., 2017)	http://www.ccpem.ac.uk/
Coot	(Emsley et al., 2010)	https://www2.mrc-lmb.cam.ac.uk/personal/pemsley/coot/
CTFFIND	(Rohou and Grigorieff, 2015)	https://grigoriefflab.janelia.org/ctf
EPU	Thermo Fisher Scientific	https://www.fei.com/software/epu/
Fiji	(Schindelin et al., 2012)	https://fiji.sc/
Gromacs 2019	(Abraham et al., 2015)	http://www.gromacs.org/
IMOD	(Kremer et al., 1996)	http://bio3d.colorado.edu/imod/
MATLAB R2017b	Mathworks	https://uk.mathworks.com/
MotionCor2 (implemented in Relion 3.0)	(Zheng et al., 2017)	N/A
novaCTF	(Turoňová et al., 2017)	https://github.com/turonova/novaCTF
PHENIX	(Adams et al., 2010)	https://www.phenix-online.org/
PRODRG	(Schüttelkopf and van Aalten, 2004)	http://www.gromacs.org/Downloads/Related_Software/PRODRG
PyMOL	(Schrödinger, 2017)	https://pymol.org/2/
REFMAC5	(Murshudov et al., 2011)	https://www2.mrc-lmb.cam.ac.uk/groups/murshudov/content/refmac/refmac.html
RELION 3.0	(Zivanov et al., 2018), (Bharat et al., 2015)	https://www2.mrc-lmb.cam.ac.uk/relion
Sequencher 5.4.6	Gene Codes Corporation	https://www.genecodes.com/
SerialEM	(Mastronarde, 2005)	http://bio3d.colorado.edu/SerialEM/
UCSF Chimera	(Pettersen et al., 2004)	https://www.cgl.ucsf.edu/chimera/
Xcalibur 4.2	Thermo Fisher Scientific	N/A

**Other**		

R2/2 200 mesh Cu/Rh holey carbon grids	Quantifoil	https://www.quantifoil.com/

### Lead Contact and Materials Availability

Further information and requests for reagents may be directed to, and will be fulfilled by the Lead Contact, Tanmay A.M. Bharat (tanmay.bharat@path.ox.ac.uk). Strains and reagents generated in this study will be made available on request, but we may require a payment and/or a completed Materials Transfer Agreement if there is potential for commercial application.

### Experimental Model and Subject Details

All *C. crescentus* strains listed in this study are listed in the Key Resources table. *C. crescentus* strains were grown in peptone-yeast-extract (PYE) medium (Poindexter, 1964) at 30°C, with antibiotic and carbon supplements at the following concentrations when necessary: kanamycin (5 μg/mL (plate), 5 μg/mL (broth)), nalidixic acid (20 μg/mL (plate)). *Escherichia coli* strains were cultured at 37°C in Luria-Bertani (LB) medium. LB medium was supplemented with kanamycin (25 μg/mL or 25 μg/mL (plate)) when necessary. Bacterial strains and plasmids used in this study are further listed in Table S2.

### Method Details

#### DNA manipulations and sequencing

All primers used in this study are listed in Table S2 and were purchased from Eurofins Genomics (Louisville, KY). PCR products were generated using iProoF Hi-Fidelity DNA polymerase (Biorad, Hercules, CA) and purified using Qiaquick spin columns (QIAGEN, Valencia, CA) following procedures recommended by the manufacturer. Chromosomal DNA was isolated using Promega Magic MiniPrep DNA purification system (Promega, Madison, WI) using the manufacturer’s instructions. DNA sequencing was performed by Eurofins Genomics. Sequence data were analyzed using Sequencher 5.4.6 software (Gene Codes Corporation, Ann Arbor, MI).

#### Construction of *rsaA* mutants

The TEV protease mutants were generated by homologous recombination using upstream and downstream fragments of *rsaA* cloned into a non-replicating plasmid pNPTS138, which carries a kanamycin resistance gene cassette (*nptI*), along with the *sacB* cassette that confers sucrose sensitivity as previously described (Gonin et al., 2000). The TEV protease site (Glu-Asn-Leu-Tyr-Phe-Gln-:-Gly) was engineered at amino acid 250 of RsaA. An upstream ∼2000 bp PCR fragment that contained 1000 bp of upstream DNA and the first 749 bp (250 aa) of *rsaA* was generated using primers, 138rsaA1kbupF and rsaATEV250upR. The downstream ∼2600 bp PCR product that contained the remainder of *rsaA* from 750 to 3080 bp (amino acid residues 251 to 1026) and an additional 500 bp of downstream DNA was created with primers, rsaATEV250dwnF and 138rsaA500dwnR. Gibson Assembly with NEBuilder HiFi Assembly mix (New England Biolabs, Ipswitch, MA) was used to assemble the two PCR products and pNPTS138 digested with EcoRV and treated with calf intestinal alkaline phosphatase (New England Biolabs). The Gibson Assembly reaction was transformed into alpha select silver chemically competent cells (Bioline, Swedesboro, NJ) and selected on LB supplemented with kanamycin. Transformants were screened by PCR using M13F, M13R and TEVprimerF and confirmed by sequencing. Plasmid (pNPTS138::3*rsaA*TEV250) was transformed into S17-1, mated into NA1000 Δ*rsaA* and selected on PYE with nalidixic acid and kanamycin. The additional selection with *sacB* was not utilized here.

The NA1000 *ΔrsaA* mutant was generated by mating pNPTS138Δ*rsaA* into NA1000, plating on PYE with kanamycin and nalidixic acid to select for the primary integrants. The transconjugants were plated on PYE sucrose and the *sacB* gene was used to select for the secondary recombination event and loss of the plasmid. The loss of *rsaA* was screen for by PCR and confirmed by sequencing.

#### Purification of RsaA_NTD_ protein

Cells from the *C. crescentus rsaA*_TEV250_ strain, containing the genomic TEV-protease cleavage site, were grown in PYE medium (Poindexter, 1964) for 24 hours at 20°C with shaking at 180 rpm. Six liters of the bacterial culture were centrifuged (5000 rcf, 4°C, 30 min). The pelleted cells were re-suspended in 50 mM HEPES/HCl buffer at pH 2.0 on ice for 10 min with vigorous shaking. Next, the suspension was centrifuged (16000 rcf, 4°C, 30 min). The pellet was discarded and the pH of the supernatant was adjusted to 7.0 with 5 M NaOH. The resulting liquid was filtered and loaded onto a 5 mL HiTrap SP HP column (GE Healthcare). All purification steps were performed using an ÄKTA pure 25 M system (GE Healthcare) operating at 4°C. The flow-through from the column was collected and dialyzed against 10 mM Tris/HCl pH 8.0 for 3 hours at 4°C. The dialyzed solution was loaded onto a 5 mL HiTrap Q HP column (GE Healthcare), washed with 20 mM Tris/HCl pH 8.0 and then eluted with the same buffer containing increasing concentrations of NaCl. Fractions containing pure RsaA_TEV250_ were collected and cleaved overnight by addition of His_6_-TEV protease in a ratio of 1:100 (wt:wt). His_6_-TEV protease was removed by loading the protein solution to a 5 mL HisTrap FF column (GE Healthcare). The flow-through was collected, concentrated and loaded to a Superdex S200 16/600 (prep grade) column (GE Healthcare) equilibrated with 25 mM HEPES/NaOH pH 7.5, 100 mM, 1 mM CaCl_2_. RsaA_NTD_ was eluted with the same buffer and fractions containing RsaA_NTD_ were collected and concentrated upto 3.7 mg/mL protein concentration (Amicon 10 kDa MWCO). Aliquots were frozen in liquid nitrogen and stored at −80°C.

#### Purification of full length RsaA

Wild-type RsaA protein was purified as described previously (Bharat et al., 2017) with modifications as follows: *C. crescentus* CB15N (NA1000) cells were grown in PYE medium for 24 hours at 25°C with shaking. The resulting culture (4 L) was centrifuged (5000 rcf, 4°C, 30 min) and the pelleted cells were re-suspended in 50 mM HEPES/HCl buffer at pH 2.0 on ice for 10 min with vigorous shaking. Next, the suspension was centrifuged (16000 rcf, 4°C, 30 min) and the cell pellet was discarded. The pH of the supernatant was adjusted to 7.0 with 5 M NaOH, filtered and loaded onto a 5 mL HiTrap SP HP column (GE Healthcare). The flow-through from the column was collected and dialyzed against 10 mM Tris/HCl pH 8.0 overnight at 4°C. The dialyzed protein solution was loaded onto a 5 mL HiTrap Q HP column (GE Healthcare), washed with 20 mM Tris/HCl pH 8.0 and then eluted with the same buffer containing increasing concentrations of NaCl. Fractions containing pure RsaA were collected and dialyzed against 20 mM Tricine/NaOH pH 8.0 and then concentrated to ∼25 mg/mL. Aliquots were flash frozen in liquid nitrogen and stored at −80°C.

#### Purification of crude LPS from *C. crescentus*

Crude LPS was purified as described previously (Jones et al., 2015), with a few modifications as follows. Crude LPS was purified from a *C. crescentus* Δ*rsaA* strain, cells were grown in PYE medium for 24 hours at 30°C with shaking at 180 rpm. Two liters of the bacterial culture were centrifuged (5000 rcf, 4°C, 30 min). The pelleted cells were washed once with 1x phosphate buffered saline (PBS) and recentrifuged (16000 rcf, 4°C, 30 min). Pelleted cells were resuspended in 60 mL of PBS supplemented with 35 mM EDTA to extract LPS from the cell surface under gentle agitation. Cells were removed by two consecutive centrifugation steps (16000 rcf, 4°C, 30 min) and resulting supernatant was treated with 50 μg/mL DNaseI and 1 U/mL benzonase (Sigma Aldrich) and dialysed against 5 mM MgCl_2_ for 4 hours at 25°C. Proteinase K was added to the dialysed solution to a final concentration of 0.01 mg/mL and incubated at 50°C overnight. The proteinase K treated sample was clarified by two centrifugation steps (50000 rcf, 4°C, 1 hour and 200000 rcf, 4°C, 3 hours). Aliquots of the resulting supernatant and pellet fractions were analyzed by sodium dodecyl sulphate-polyacrylamide gel electrophoresis (SDS-PAGE) and stained with Pro-Q Emerald 300 Lipopolysaccharide Gel Stain Kit (ThermoFisher). While a minor fraction of the isolated LPS was lost in the pellet, the majority was found in the supernatant which was concentrated 120 times (Amicon 3.5 kDa MWCO) and loaded onto a Superose 6 10/300 GL column (GE Healthcare) equilibrated with 200 mM NaCl. Crude LPS was eluted with the same buffer and monitored at a wavelength of 215 nm. Fractions containing high absorbance were collected and analyzed by SDS-PAGE and Pro-Q Emerald 300 staining and Coomassie brilliant blue G-250. Aliquots containing crude LPS were flash frozen in liquid nitrogen and stored at −80°C.

#### Reconstitution of the RsaA_NTD_:PS complex

PS was partially released from purified, crude LPS by hydrolysis with acetic acid (1% (v/v), 95°C, 2 hours). The sample was clarified by centrifugation (16000 rcf, 4°C, 30 min) and adjusted to pH 7.0 by addition of 1 M HEPES/NaOH pH 7.0. An excess of purified RsaA_NTD_ was mixed with hydrolysed PS and the mixture was dialyzed against 25 mM HEPES/NaOH pH 7.5, 100 mM NaCl, 1mM MgCl_2_, 1mM CaCl_2_ overnight at 4°C. The sample was loaded to a Superose 6 10/300 GL column (GE Healthcare) equilibrated with the same buffer. Peak fractions containing oligomeric RsaA_NTD_ were collected, concentrated (Amicon 30 kDa MWCO) and flash frozen in liquid nitrogen and stored at −80°C.

#### Preparation of samples for cellular cryo-ET

For cellular cryo-ET (Figure 4), CB15N *C. crescentus* cells or cells with genomic deletion of *rsaA* (Δ*rsaA* strain), were grown in PYE medium to late log phase at 30°C with vigorous shaking and directly used for grid preparations. For probing binding of RsaA to cellular LPS, Δ*rsaA* cells were mixed with purified RsaA_NTD_ (∼1 mg/mL final concentration) or with RsaA (full-length) protein (∼2.5 mg/mL final concentration). Cells were incubated with vigorous shaking for 15 min at room temperature before vitrification. Ca^2+^ dependence of RsaA_NTD_ binding to the cell surfaces was tested by centrifuging 5 mL of late log phase Δ*rsaA* cells (16000 rcf, 25°C, 10 min) and resuspending the pellet in the 5 mL of liquid containing 10 mM HEPES pH 7.5, 5 mM ethylene glycol bis(2-aminoethyl ether)-*N*,*N*,*N*′,*N*′-tetraacetic acid (EGTA). This process was repeated three times, and the sample was mixed with purified RsaA_NTD_ (∼1 mg/mL final concentration) and incubated at room temperature with vigorous shaking for 15 min before vitrification.

#### Cryo-EM and cryo-ET sample preparation

For cryo-EM grid preparation 2.5 μL of purified RsaA_NTD_ or RsaA_NTD_:PS complex (2.5 mg/mL) was applied to a freshly glow discharged Quantifoil R2/2 Cu/Rh 200 mesh grid, adsorbed for 10 s, blotted for 3 s and plunge-frozen into liquid ethane in a Vitrobot Mark IV (ThermoFisher), while the blotting chamber was maintained at 100% humidity at 10°C. For cryo-ET sample preparation, purified or cellular samples were mixed with 10 nm protein-A gold (CMC Utrecht) before application to the cryo-EM grid.

#### Cryo-EM and cryo-ET data collection

Single-particle cryo-EM data was collected on a Titan Krios G3 microscope (ThermoFisher) operating at 300 kV fitted with a Quantum energy filter (slit width 20 eV) and a K2 direct electron detector (Gatan) with a sampling pixel size of 1.08 Å running in counting mode. In total 2422 movies were collected with a dose rate of 6.3 e^-^/pixel/s on the camera level. The sample was subjected to 8 s of exposure where a total dose of 43 e^-^/Å^2^ was applied, and 20 frames were recorded per movie. Data collection for initial model generation using sub-tomogram was performed on the same Titan Krios microscope using the Quantum energy filter (slit width 30 eV) and the K2 direct electron detector running in counting mode with a dose rate of 5.5 e^-^/pixel/s (Gatan). Tilt series (6 in total) with a defocus range of −3 to −6 μm were collected between ± 60° in two directions from 0° at 2° tilt increment. A total dose of 100 e^-^/Å^2^ was applied over the entire series, and image data was sampled at a calibrated pixel size of 2.238 Å. For cellular samples, tilt series (wt: 10 tilt series; Δ*rsaA*: 13 tilt series; Δ*rsaA* + RsaA_NTD_: 7 tilt series; Δ*rsaA* + RsaA: 2 tilt series) were collected at a dose rate of 11.3 e^-^/pixel/s, with a total dose over the series of 81 e^-^/Å^2^. Data was sampled at a calibrated pixel size of 5.571 Å using the K2 direct electron detector running in counting mode (slit width 20 eV). For high-resolution *in situ* structure determination of the S-layer, a pipeline for high-throughput data collection was adopted (Wan et al., 2017). Briefly, a Titan Krios microscope was used to collect tilt series data with a dose symmetric tilting scheme (Hagen et al., 2017). Tilt series were collected at a pixel size of 1.3 Å, with a total dose of 140 e^-^/Å^2^ was applied over entire series collected between ± 60° with 3° tilt increments (Bharat et al., 2017).

#### Cryo-EM single-particle image processing

Initial model generation from cryo-ET data was performed using the Relion sub-tomogram averaging pipeline (Bharat et al., 2015, Bharat and Scheres, 2016). An unambiguous 3D reference was generated and used in the single-particle EM pipeline as follows. Movies were motion corrected and dose weighted with MotionCor2 (Zheng et al., 2017) implemented in Relion 3.0 (Zivanov et al., 2018). Contrast transfer functions (CTFs) of the resulting motion corrected micrographs were estimated using CTFFIND4 (Rohou and Grigorieff, 2015). Particles were extracted with a 300 pixel × 300 pixel box and classified using reference-free 2D-classification inside Relion 3.0. Particles from classes showing high-resolution features were subjected to 3D classification using the unbiased sub-tomogram averaging reference structure described above. Particles from two main 3D classes containing 21 or 20 RsaA_NTD_ subunits were combined for a focused 3D auto refinement on the central 14 subunits using the output from the 3D classification as a starting model. The final map was obtained from 115,776 particles and post-processed using a soft mask focused on the inner fourteen subunits yielding a sharpened map with a *B*-factor of −85.8 Å^2^ and a resolution of 3.68 Å according to the gold standard Fourier shell correlation criterion of 0.143 (Scheres and Chen, 2012).

#### Sub-tomogram averaging

Tilt series alignment using gold fiducials and tomogram generation was carried out using IMOD (Kremer et al., 1996). Sub-tomogram averaging processing was performed using custom scripts written in MATLAB, described in detail elsewhere (Bharat et al., 2011, Wan et al., 2017). Cellular sub-tomogram averages (see Figures 4 and S5) were lowpass filtered to the same resolution of 40 Å for comparison. For high-resolution cryo-ET structure determination, we adopted previously published methods (Bharat et al., 2017) with the major difference being the use of a recently developed in 3D-CTF correction method for tomographic data (Turoňová et al., 2017). In addition, we used focused alignment on the RsaA_NTD_ part of the S-layer to improve this region of the map to observe LPS binding. The final map was obtained from 51,866 hexameric units of the S-layer from 110 tomograms (Bharat et al., 2017). Post-processing using a softened mask focused on the inner domain and pore region of RsaA yielded a sharpened map with a *B*-factor of −224.953 Å^2^ applied and a resolution of 4.82 Å (Figure S6) according to the 0.143 criterion. The final map has not been explicitly symmetrized and local resolution differences were estimated in Relion 3.0. Figures were prepared using Fiji (Schindelin et al., 2012) and MATLAB.

#### Model building and refinement

The carbon backbone of the RsaA_NTD_ protein was manually traced through a single subunit of the cryo-EM density using Coot (Emsley et al., 2010). Initially, side chains were assigned in regions with density corresponding to characteristic aromatic residues allowing us to deduce the register of the amino acid sequence in the map. Side chains for residues 2-243 of RsaA were thus assigned unambiguously and the structure was refined and manually rebuilt using Refmac5 (Murshudov et al., 2011) inside the CCP-EM (Burnley et al., 2017) software suite and Coot. Additional subunits around the spiral of the RsaA_NTD_:PS complex were generated by rigid body fitting the refined monomeric unit into the cryo-EM density. Areas of strong and continuous density connecting protein subunits along the long axis of the spiral could not be explained by any amino acid residues in RsaA_NTD_ and were therefore assigned to the O-antigen of the *C. crescentus* LPS, to which RsaA_NTD_ is known to bind (Bharat et al., 2017, Ford et al., 2007). The chemistry of the heptameric repeating unit of the O-antigen has been described previously (Jones et al., 2015), and was used to build the main chain of the PS. Orientation of the PS was assigned by iterative rebuilding and refinement using restraints for N-Acetyl-perosamine (PerNac) which were generated with PRODRG (Schüttelkopf and van Aalten, 2004) and existing restraints for mannose. Comprehensive model validation of the final structure and map was performed in PHENIX (Adams et al., 2010) (see Table S1). The final refined cryo-EM structure of RsaA_NTD_ and the X-ray structure of RsaA_CTD_ (PDB ID 5N8P) were rigid body docked into the 4.8 Å cryo-ET map and were not refined further. Figures containing protein structures or cryo-EM/ET data were prepared using USCF Chimera (Pettersen et al., 2004).

#### Native mass spectrometry

Samples were loaded into a gold coated needle prepared in-house and introduced into a Q-Exactive UHMR mass spectrometer (ThermoFisher), as described previously (Gault et al., 2016). The following parameters were used: capillary voltage was set to 1.2-1.4 kV, resolution was set to 17500 at m/z 200, injection flatopole was set to 5 V, inter flatopole lens was at 4 V, and bent flatopole at 2 V. Backing pressure was maintained at ∼3 × 10^−9^ mbar. Stripped oligomers were obtained by MS/MS analysis at a voltage applied to the HCD cell of 220 V. Zeba™ micro spin desalting columns were used to remove the Ca^2+^ ions. Data was analyzed using Xcalibur 4.2.

#### Molecular dynamics simulations

Atomistic simulations were run in triplicate for 100 ns using the CHARMM36m forcefield (Huang et al., 2017). Simulations were performed at 310 K using the velocity-rescaling temperature coupling algorithm (Bussi et al., 2007), with a time constant of 0.1 ps and Parrinello-Rahman isotropic pressure coupling of 1 bar with a time constant of 2 ps (Parrinello and Rahman, 1981). Electrostatics were handled using the Particle-Mesh-Ewald method (Darden et al., 1993), and a force-switch modifier was applied to the Van der Waals forces. Dispersion corrections were turned off. The parameters for the O-antigen were generated using the CHARMM-GUI (Kim et al., 2017, Lee et al., 2016). All simulations were run using Gromacs 2019 (Abraham et al., 2015). Molecular simulation images and Supplemental Movies of simulations were made in PyMOL (Schrödinger, 2017). Graphs were plotted using Python and Matplotlib.

### Quantification and Statistical Analysis

See METHOD DETAILS for further information on the statistical analyses including replicates for MD simulations and resolution estimates for cryo-EM maps.

### Data and Code Availability

#### Data resources

The cryo-EM map of RsaA_NTD_:PS complex together with the build atomic model have been deposited in the Electron Microscopy Data Bank (EMDB) with the accession code EMD-10389 and the Protein Data Bank (PDB) with accession code 6T72 respectively. The cryo-ET map of the native S-layer has been deposited with the EMDB accession code EMD-10388.

#### Software

All software used in this study has been extensively described in previous publications from our and other laboratories. See the METHOD DETAILS section for citations to the original publications.
