# Supplementary material for: In Situ Structure of an Intact Lipopolysaccharide-Bound Bacterial Surface Layer
Source: Cell. 2020 Jan 23;180(2):348–358.e15. doi: 10.1016/j.cell.2019.12.006 (PMC6978808; doi:10.1016/j.cell.2019.12.006)
Supplement: Document S1. Tables S1 and S2 [file mmc1.pdf]

Cell, Volume *180*

## Supplemental Information

# *In Situ* Structure of an Intact Lipopolysaccharide-Bound Bacterial Surface Layer

**Andriko von Kügelgen, Haiping Tang, Gail G. Hardy, Danguole Kureisaite-Ciziene, Yves V. Brun, Phillip J. Stansfeld, Carol V. Robinson, and Tanmay A.M. Bharat**

## Supplemental Tables

**Table S1: Cryo-EM data collection, refinement and validation statistics, Related to Figure 1.**

| | #$RsaA_{NTD}$:PS(EMDB: EMD-10389), (PDB: 6T72) |
|---|---|
| **Data collection and processing** | |
| Magnification | 130,000 |
| Voltage (kV) | 300 |
| Electron exposure (e–/Å$^2$) | 43 |
| Defocus range (μm) | -1.5 to -3 |
| Pixel size (Å) | 1.08 |
| Symmetry imposed | C1 |
| Initial particle images (no.) | 129633 |
| Final particle images (no.) | 115776 |
| Map resolution (Å) | 3.7 |
| FSC threshold | 0.143 |
| Map resolution range (Å) | 3.68-4.5 |
| | |
| **Refinement** | |
| Initial model used (PDB code) | None |
| Model resolution (Å) | 3.7 |
| FSC threshold | 0.5 |
| Model resolution range (Å) | 3.7-4.5 |
| Map sharpening *B* factor (Å$^2$) | -85.819 |
| Model composition | |
| Non-hydrogen atoms | 26278 |
| Protein residues | 3388 |
| Ligands | 210 |
| *B* factors (Å$^2$) | |
| Protein | 70.97 |
| Ligand | 147.87 |
| R.m.s. deviations | |
| Bond lengths (Å) | 0.007 |
| Bond angles (°) | 1.515 |
| Validation | |
| MolProbity score | 1.23 |
| Clashscore | 4.29 |
| Poor rotamers (%) | 0.00 |
| Ramachandran plot | |
| Favored (%) | 97.92 |
| Allowed (%) | 2.08 |
| Disallowed (%) | 0.00 |

*based on 14 $RsaA_{NTD}$ subunits

**Table S2: Strains, plasmids and oligos used in this study, Related to Method Details.**

| Strain, plasmid or oligo | Description, construction or sequence (5' – 3') | Reference or application |
|---|---|---|
| **Strains** | | |
| *Escherichia coli* | | |
| S17-1 | *E. coli* 294::RP4-2(Tc::Mu)(KM::Tn*7*) | (Simon et al., 1983) |
| Alpha-select™ | *deoR endA1 recA1 relA1 gyrA96 hsdR17*($r_k^-$ $m_k^+$) *supE44 thi*-1 Δ(*lacZYA-argFV169*) Φ80δ*lacZ*Δ*M15* $F^-$ | Bioline |
| *Caulobacter crescentus* | | |
| NA1000 | *syn-1000*; previously called CB15N. This strain does not make the adhesive holdfast. | (Evinger and Agabian, 1977) |
| YB5754 | NA1000 Δ*rsaA* | E. Quardokus, unpublished |
| YB1001 | NA1000 Δ*rsaA* ::pNPTS138::*rsaA*TEV250 | This study |
| **Plasmids** | | |
| pNPTS138 | pLitmus 38 derivative; with *nptI, sacB* and RK2 *oriT* sequences, and deleted *bla* gene; $Km^R$ | M.R.K. Alley, unpublished |
| pNPTS138::*rsaA*TEV250 | pNPTS138 parent vector containing 1000 bp fragment upstream of *rsaA*, *rsaA* with TEV protease site inserted at amino acid residue 250 and 500 bp downstream of *rsaA* | This study |
| pNPTS138Δ*rsaA* | A 471 bp upstream of *rsaA* and a 530 bp PCR product downstream of *rsaA* were cloned into pNPTS138. | (Hardy et al., 2010) |
| **Oligo Name** | | |
| 138rsaA1kbupF | AGCTTCTCTGCAGGATATTGGCCGGCAGTTGCACCG | *rsaA*TEV250 construct |
| 138rsaA500dwnR | AATTCGTGGATCCAGATGGGCGGGCGACCAGCACG | *rsaA*TEV250 construct |
| rsaATEV250upR | TGGAAGTACAGGTTCTCCGAACCCGACACGCCCGA | *rsaA*TEV250 construct |
| rsaATEV250dwnF | CGTGTCGGGTTCGGAGAACCTGTACTTCCAGGGCA CCCTCTCGCTGACCACCGGC | *rsaA*TEV250 construct |
| 662uprsaAF | TGGCTCCAGTCCGCCGGTTGC | *rsaA* sequencing primer |
| 132uprsaAF | TATAGCGCTTTTCGGCGGGGG | *rsaA* sequencing primer |
| rsaA338F | CTACTCGAAGTTCGCTCAGG | *rsaA* sequencing primer |
| rsaA842F | CACCGCCAACAACGACACG | *rsaA* sequencing primer |
| rsaA1338F | GTGGCTCAAACGGCCGGC | *rsaA* sequencing primer |
| rsaA1838F | CGCTCGCGTCACGATCACCTC | *rsaA* sequencing primer |
| rsaA2338F | CGTGTTCAACCTGACCCTGTC | *rsaA* sequencing primer |
| rsaA2834F | GACAAGCTCGACCTCGTC | *rsaA* sequencing primer |
| 564uprsaAR | CACCTAGGCTTGGGGAAGCTTCCG | *rsaA* sequencing primer |
| 62uprsaAR | CAGCATTTTTCTAACCGGTACAGC | *rsaA* sequencing primer |
| rsaA447R | ACGAAACGCCCGTGTAGG | *rsaA* sequencing primer |
| rsaA942R | CAGGACGTCGGTGCCAGC | *rsaA* sequencing primer |
| rsaA1445R | GGCGGCGGTTTGGGTGAC | *rsaA* sequencing primer |
| rsaA1939R | AGACCGGTCGCCAGTTCG | *rsaA* sequencing primer |
| rsaA2445R | CGGTCGTGTTGGTGTCGG | *rsaA* sequencing primer |
| rsaA2938R | AGGTACTGAGCCAGGGTC | *rsaA* sequencing primer |
| FHindIIIrsaA | GACCTCCAGAAGCTTGGCCCAGTC | ΔrsaA PCR/sequencing |
| RsalIrsaA | GCCGTCGAAGTCGAGACCGCC | ΔrsaA PCR/sequencing |
| TEVprimerF | GAGAACTTGTACTTCCAGTCG | TEV protease site primer |
| M13R(-48) | AGCGGATAACAATTTCACACAGGA | pNPTS138 PCR/sequencing |
| M13F (-40) | GTTTTCCCAGTCACGAC | pNPTS138 PCR/sequencing |
